# Butyrate ameliorates maternal high-fat diet-induced fetal liver cellular apoptosis

**DOI:** 10.1371/journal.pone.0270657

**Published:** 2022-07-06

**Authors:** Yu-Jyun Huang, Pei-Ming Wang, Kuo-Shu Tang, Chih-Jen Chen, Ying-Hsien Huang, Mao-Meng Tiao

**Affiliations:** 1 Department of Pediatrics, Kaohsiung Chang Gung Memorial Hospital, and Chang Gung University College of Medicine, Kaohsiung, Taiwan; 2 Department of Family Medicine, Kaohsiung Chang Gung Memorial Hospital, and Chang Gung University College of Medicine, Kaohsiung, Taiwan; Universite du Quebec a Montreal, CANADA

## Abstract

A maternal high-fat diet (HFD) can impact the offspring’s development of liver steatosis, with fetal development in utero being a crucial period. Therefore, this study investigated the mechanism and whether butyrate can rescue liver injury caused by maternal HFD in the fetus. Pregnant female Sprague Dawley rats were randomly divided into two groups, prenatal HFD (58% fat) exposure or normal control diet (4.5% fat). The HFD group was fed an HFD 7 weeks before mating and during gestation until sacrifice at gestation 21 days. After confirmation of mating, the other HFD group was supplemented with sodium butyrate (HFSB). The results showed that maternal liver histology showed lipid accumulation with steatosis and shortened ileum villi in HFD, which was ameliorated in the HFSB group (P<0.05). There was increased fetal liver and ileum TUNEL staining and IL-6 expression with increased fetal liver TNF-α and malondialdehyde expression in the HFD group (P<0.05), which decreased in the HFSB group (P<0.05). The fetal liver expression of phospho-AKT/AKT and GPX1 decreased in the HFD group but increased in the HFSB group (P<0.05). In conclusion that oxidative stress with inflammation and apoptosis plays a vital role after maternal HFD in the fetus liver that can be ameliorated with butyrate supplementation.

## Introduction

The developmental origin of health and disease is an important issue [1]. The fetus in particular is exposed to a wide range of environmental conditions, including over-nutrition [2]. Fetal development in utero is a particularly vulnerable time during which the maternal environment affects long-term fetal growth and disease development [3]. Indeed, nonalcoholic fatty liver disease can originate in early life and lead to offspring metabolic syndrome, liver cirrhosis, and end-stage liver disease [4]. Moreover, the deleterious effects of a high-fat diet (HFD) on perinatal can impair adult liver [5], so it was hypothesized that maternal HFD during pregnancy results in liver steatosis and retroperitoneal adiposity in the offspring [6, 7].

Early maternal diets can influence gut microbiota composition in the offspring through *in utero* maternal gut microbiota transfer [2, 8]. Furthermore, maternal HFD during pregnancy induces dysbiosis in the offspring [8], with these changes persisting until adulthood with long-term effects on metabolic fatty liver development [9]. The gut microbiota metabolite, short chain fatty acid butyrate, produced by the microbial fermentation of dietary fiber in the large intestine has multiple beneficial effects in mammals [10, 11]. Butyrate improves glucose tolerance in the rat liver by inhibiting inflammation, reducing steatosis and metabolic syndrome [12, 13]. It decreased malondialdehyde (MDA) with cellular oxidative stress to prevent liver toxicity [14], as well as increasing antioxidative enzyme activities to ameliorate liver oxidative damage and several pro-inflammatory genes [15, 16]. The rationale for the study is that oxidative stress in the liver damage induced by a maternal HFD could be recovered by butyrate.

The implications of prenatal insults on the risk of developing a disease in offspring provide a basis for the study of the development of liver steatosis in the fetus. The mechanisms by which fetal metabolic systems cope with excess nutrition during intrauterine development are important and remain relatively unexplored. Hence, this study explored whether butyrate therapy can inverse prenatal HFD stress in the fetus to provide insights for future clinical management strategies.

## Materials and methods

### 2.1 Animals

The animal study was performed at the Animal Experimental Center of Kaohsiung Chang Gung Memorial Hospital after approval from the Institutional Animal Care and Use Committee of the Hospital (Approval No. 2019052802). Sprague Dawley (SD) rats were purchased from Lasco Laboratories (Taipei, Taiwan) and were housed in the animal facility in a 12-hr light/dark cycle with lights on at 7 a.m., and litters were checked at 10:00 am daily.

The HFD is composed of 5.56 kcal/g dry weight, 23 g/100 g protein, 35.5 g/100 g carbohydrate, and 35.8 g/100 g saturated fat (58 kcal% fat D12331) was purchased from the Research Diet Company, USA. The normal-chow diet (NCD) is composed of 3.85 kcal/g dry weight, 19.2 g/100 g protein, 67.3 g/100 g carbohydrate, and 4.3 g/100 g saturated fat. The female rats were allowed 24 h to mate with male rats, then separated from the male rats and housed individually in a standard plastic cage.

Sprague Dawley rats and their fetuses were randomly divided into groups, prenatal HFD, NCD, and HFSB (N = 6 for each group). Each group of two pregnant female rats will give birth to an average of 20 offspring. Group I NCD: SD rats were fed with an NCD before mating and during gestation till sacrifice on gestational day 21 (GD21). Group II HFD: Maternal SD rats were fed with HFD 7 weeks before mating and during gestation till sacrifice on GD21. Group III HFSB: Maternal SD rats were fed with HFD 7 weeks before mating and during gestation and the pregnant rats were administrated with 1% (w/v) sodium butyrate (B5887, SIGMA, MO, USA) in drinking water (150 mg/kg/day) via gavage [17] from GD0 until sacrifice at GD21. There were no adverse effects on gastrointestinal symptoms observed during sodium butyrate administration.

### 2.2 Measurement of plasma biochemistry parameters

Blood samples were collected by cardiac puncture when the rats were sacrificed to alleviate their suffering [18]. The plasma levels of total cholesterol, aspartate transaminase (AST), and alanine aminotransferase (ALT) were determined by a standard auto-analyzer (Hitachi model 7450, Tokyo, Japan).

### 2.3 Tissue preparation

Rats were anesthetized with 25 mg/kg Zoletil and 23 mg/kg Xylazine on GD21 until no conscious then perfused continuously with normal saline via a peristaltic pump. The maternal liver and ileum were immediately removed and kept on an ice plate. The lumen of the small intestinal tissues was cleaned using ice-cold phosphate-buffered saline solution (PBS, pH 7.4) and embedded in Swiss rolls in paraffin, followed by hematoxylin and eosin (H&E) staining for evaluation of the villi length. The liver tissue was cut into pieces and embedded in paraffin for immunohistochemistry. The remainder of the liver and ileum were dissected and stored at -80°C. The fetal liver and ileum were also collected for analysis. Though endogenous butyrate absorption by colonocytes have been well documented [19], the effect of maternal butyrate intake on the fetal ileum and liver is unknown. We therefore studied both fetal ileum and liver tissue.

### 2.4 H&E staining (hematoxylin and eosin)

The liver and small intestine were dissected and fixed in 4% paraformaldehyde at 4°C overnight, then dehydrated in a gradient of ethanol (70%, 75%, 85%, 90%, 95%, and 100%), hyalinized in xylene, and embedded in paraffin wax at 55°C. Sections (4 μm thick) were cut and stained with an H&E Staining Kit (ScyTek Laboratories, West Logan, USA). The histologic lesions were observed with a Leica DMI-3000 microscope equipped with a digital camera and lipid accumulation in the liver and length villus of ileum were quantified using ImageJ (Fiji version 1.8.0) [20]. The semi-quantitation of the lipid droplets was calculated using approximately 500 liver cells.

### 2.5 Gas chromatography-mass spectrometry (GC-MS) analysis of short chain fatty acids (SCFAs)

SCFAs in stool samples were quantified and analyzed by GC-MS (Agilent GC system 7890B, MSD system 5977B). Briefly, 15 μl of stool was acidified with 50 μl of 50% sulfuric acid and 10 μl of 2-ethylbutyric acid as internal standard, before the addition of 400 μl ether and shaken for 15 min, followed by centrifugation at 9000 rpm at 4°C for 10 min. The ether layer was removed and mixed with anhydrous sodium sulfate for dehydration before 1 μl of the sample was injected (split ratio: 5:1) into a straight glass liner and held at 240°C. Helium (1 ml/min) was used as a carrier gas in the DB-FFAP capillary column (30 m×0.25 mm×0.25 μm). The oven temperature of 80°C was initially maintained for 1 min, then increased to 150°C at a rate of 5°C/min, finally increased to 240°C at a rate of 10°C/min for 12 min. The electron ionization (70 eV and 230°C) mode was used, with all samples, standards, and blanks analyzed randomly.

### 2.6 Immunohistochemistry

The 4 μm-sections of formalin-fixed tissues were mounted on silanized slides, deparaffinized in xylene, and rehydrated through serial baths of alcohol to water. To eliminate endogenous peroxidase activity, the hydrated sections were treated with 3% hydrogen peroxide for 10 mins and washed in PBS. Anti-IL-6 antibody (ab6672, Abcam; Cambridge, MA, USA) was diluted 1:200 in DaVinci Green (Biocare PD900, CA). The assay was assessed by an independent researcher (YMC) and one of the authors (MMT) using the Ultravision Quanto Detection system HRP DAB kit (Thermo Scientific In., TL-060-QHD; Waltham, MA, USA). The samples were observed with a Leica DMI-3000 microscope equipped with a digital camera and staining quantified by the ImageJ (Fiji version 1.8.0) [20].

### 2.7 Terminal deoxynucleotidyl transferase-mediated deoxyuridine triphosphate biotin nick-end labeling (TUNEL)

Fixed tissues embedded in paraffin were cut into 4 μm sections and mounted on slides. Apoptosis was assessed using an apoptosis detection kit (Roche, 11684817910, Mannheim, Germany) according to the manufacturer’s instructions [7]. Cells were counted from randomly selected high-power fields (200x, 400x) from each section under light microscopy. The rates of TUNEL-positive cells were calculated using 10 random fields from each rat counted for positively stained cells in the liver and each villus of the ileum.

### 2.8 Western blotting

Liver and ileum specimens were homogenized in lysis buffer (iNtRON, 17081; Biotechnology, Seongnam, Korea) and centrifuged at 14,000×g at 4°C for 5 min. The total protein concentration was evaluated using Bio-Rad Protein Assay Dye Reagent Concentrate (Bio-Rad Laboratories, Inc., Hercules, CA, USA). Protein samples (65 μg) were separated by 6~15% sodium dodecyl sulfate polyacrylamide gel electrophoresis and transferred to polyvinylidene difluoride membranes. Membranes were blocked in TBST buffer with 10% non-fat milk for 1 h at room temperature, before incubation at 4°C overnight with specific primary antibodies including 1:2000 tumor necrosis factor-alpha (TNF-α, #3707; Cell Signaling, Denver, MA, USA), 1:1000 cleaved-caspase 3 (#9661, Cell Signaling, Denver, MA, USA), 1:1000 phospho-AKT (Ser 473) (#9271, Cell Signaling, Denver, MA, USA), 1:1000 AKT (#9272, Cell Signaling, Denver, MA, USA), 1:1000 Zonula occludens-1 (ZO-1) (67–7300; Thermo Fisher Scientific Inc., Waltham, MA, USA), 1:1000 Claudin-3 (ab15102; Abcam, Cambridge, MA, USA), 1:1000 Occludin (#91131, Cell Signaling, Denver, MA, USA), 1:1000 glutathione peroxidase 1 (*GPX1)* (ab22604, Abcam, Cambridge, MA, USA) and 1:2000 Malondialdehyde (MDA) (ab27642, Abcam, Cambridge, MA, USA), 1:5000 GAPDH (ab181602, Abcam, Cambridge, MA, USA), 1:10000 beta actin (MAB1501, Merck Millipore, Temecula, CA, USA). The membranes were incubated with secondary horseradish peroxidase-conjugated anti-rabbit antibody (1:5000, Jackson ImmunoResearch, West Grove, PA USA) or anti-mouse antibody (1:10000, Jackson ImmunoResearch, West Grove, PA USA) for 1 h at room temperature, before visualization using an ECL kit (Perkin Elmer In., NEL 105001EA; Boston, MA, USA).

### 2.9 Statistical analysis

Data were expressed as mean ± standard error of the mean. Continuous data of biochemical parameters (serum AST, ALT, total cholesterol, maternal and fetal body weight, liver weight), western blot and histology results were analyzed by one-way ANOVA with Tukey post-hoc tests, with a P-value <0.05 considered statistically significant. All analyses were performed using the Statistical Package for the Social Sciences (SPSS) software version 12.

## Results

### Maternal weight, biochemical data, and fetal weight

The pregnant rat body weight, maternal net weight, and fetal body weight were all increased in the HFD group (P<0.001, P<0.001, P = 0.021, respectively) and decreased in HFSB (P = 0.002, P = 0.002, P = 0.019, respectively). The maternal net weight was calculated by subtracting the fetal weight and placental weight from the maternal weight. Maternal liver weight gain occurred in the HFD group but was not reversed in the HFSB group. The AST level increased in the HFD (P = 0.027) and decreased in the HFSB group (P = 0.008), whereas ALT was not significantly different among the three groups. The total cholesterol level was unchanged in HFD rats but decreased in the HFSB group (P = 0.042) (Table 1).

**Table 1:** Maternal weights, biochemical data and fetal weight of the studied groups.

	BW (g)	Liver (g)	Liver / BW	Maternal net weight (g)	Fetal BW (g)	AST (U/L)	ALT (U/L)	T-Cholesterol (mg/dL)
**NCD**	327.6 ± 6.6^#^^†^	8.60 ± 0.3^#^^†^	0.026 ± 0.01^#^^†^	288.5 ± 6.3^#^^†^	2.83 ± 0.25^#^^†^	85.9 ± 10.3^#^^†^	34.6 ± 1.6	62.6 ± 7.3^†^
**HFD**	405.1 ± 11.4*^†^	13.77 ± 0.6*	0.03 ± 0.02*	355.0 ± 12.2*^†^	3.35 ± 0.42*^†^	100.6 ± 9.2*^†^	34.4 ± 2.0	60.7 ± 8.1^†^
**HFSB**	373.1 ± 10.1*^#^	12.12 ± 0.7*	0.03 ± 0.02*	311.1 ± 7.8*^#^	2.75 ± 0.16*^#^	79.3 ± 10.5*^#^	34.8 ± 2.2	50.1 ± 6.7*^#^

Data were presented as mean ± standard error.

AST: aspartate transaminase, ALT: alanine aminotransferase, BW: body weight, Maternal net weight: calculated by subtraction of fetal weight and placental weight from maternal weight, T-cholesterol: total cholesterol, NCD: normal-chow diet, HFD: high-fat diet, HFSB: high-fat diet co-treated with butyrate during gestation

* p < 0.05 compared to NCD;

^#^ p < 0.05 compared to HFD;

^†^ p < 0.05 compared to HFSB treated.

### Maternal liver and ileum histology

**Fig 1:**
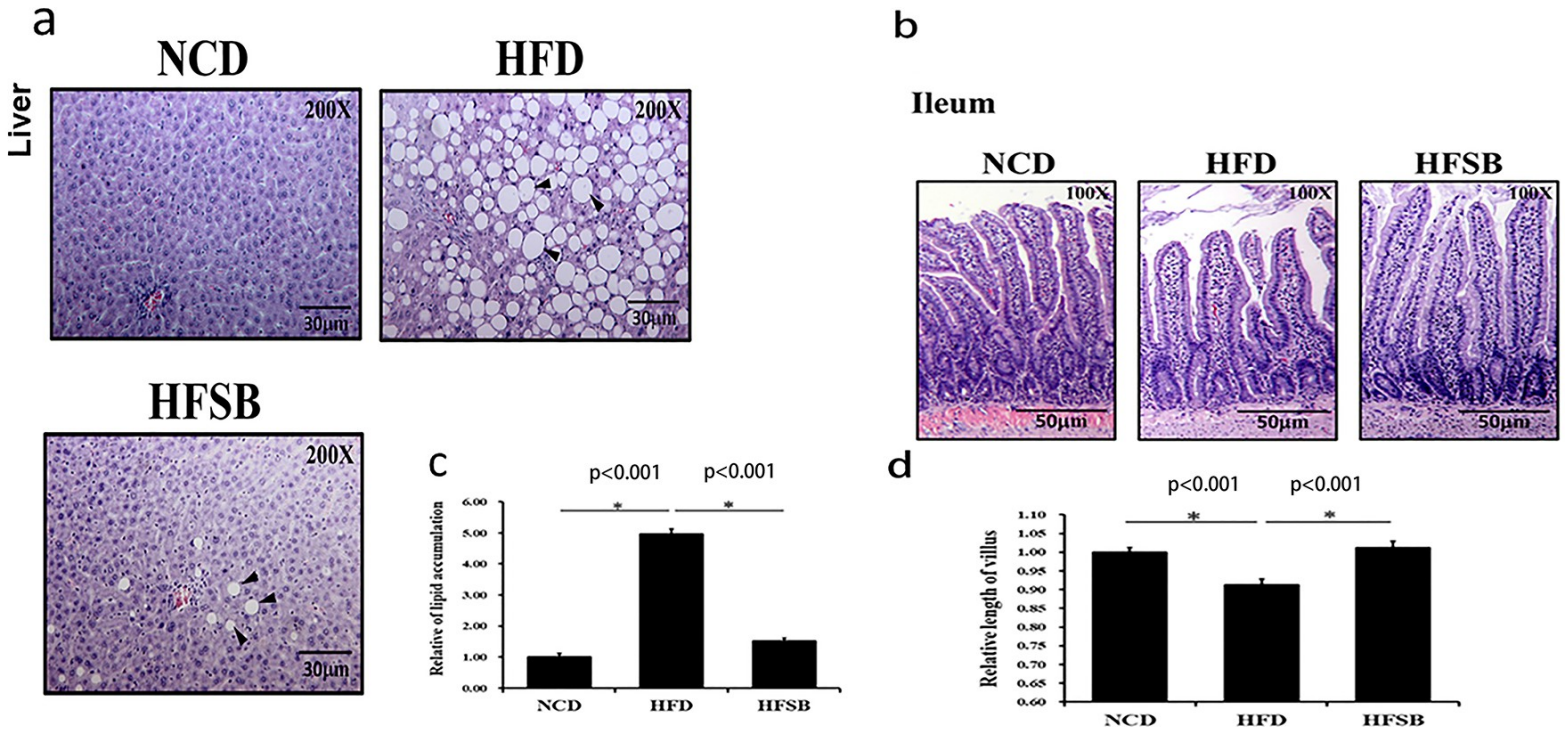
Histological analysis of maternal liver and ileum. (a) H&E staining in the liver and (b) ileal tissue. Semi-quantitative analysis of (c) hepatic lipid accumulation and (d) ileal villous length. NCD: normal-chow diet, HFD: high-fat diet, HFSB: high-fat diet with butyrate supplementation during pregnancy (n = 6), * p < 0.05. Black arrowheads: lipid droplets.

The liver histology of pregnant rats demonstrated steatosis with increased lipid accumulation (lipid droplets) in the HFD group, whereas there was decreased lipid accumulation in HFSB (Fig 1a and 1c) The shortened ileum villi with maternal HFD in pregnant rats recovered in the HFSB group (Fig 1b and 1d).

### Maternal stool SCFAs

**Fig 2:**
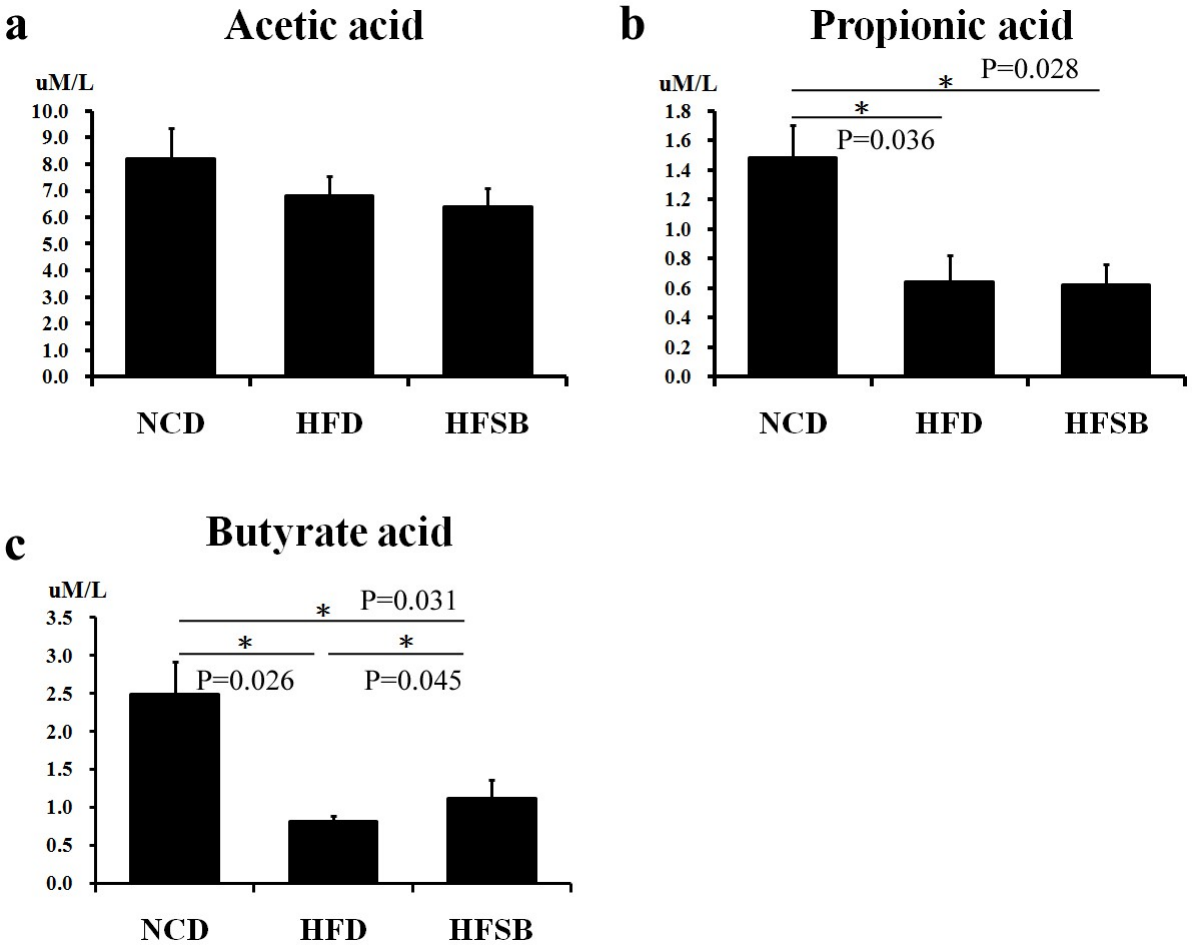
Short chain fatty acids profile of maternal rats. Maternal stool levels of (a) acetic acid, (b) propionic acid, (c) and butyric acid. NCD: normal-chow diet, HFD: high-fat diet, HFSB: high-fat diet with butyrate supplementation during pregnancy (n = 6), * p < 0.05.

There was no significant difference among the three groups regarding maternal stool levels of acetic acid levels (Fig 2a). The maternal stool levels of propionic acid and butyric acid decreased in HFD (Fig 2b and 2c), while only butyric acid increased in HFSB (Fig 2c).

### Fetal liver and ileum inflammation

**Fig 3:**
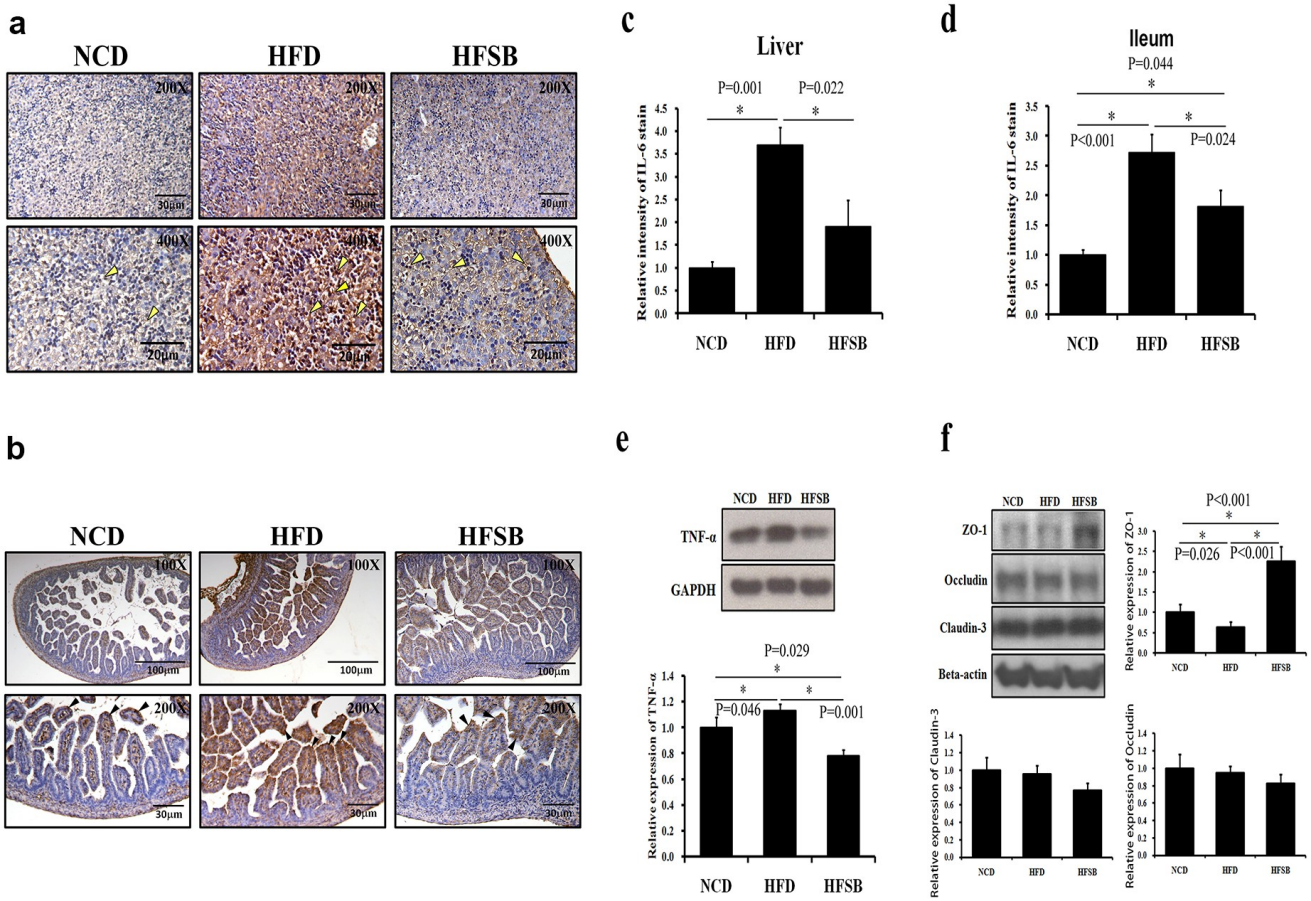
Fetal liver and ileum inflammation. (a) IL-6 staining in fetal liver and (b) fetal ileum. (c, d) Semi-quantitative analysis of IL-6 staining in fetal liver and fetal ileum, (e) western blotting of TNF-α expression in fetal liver, and (f) western blotting of fetal ileum tight junction ZO-1, occludin and claudin-3 expressions. NCD: normal-chow diet, HFD: high-fat diet, HFSB: high-fat diet with butyrate supplementation during pregnancy (n = 6), * p < 0.05. Arrowheads: positive IL-6 staining cells.

IL-6 and TNF-α was used as inflammation markers [21]. IL-6 staining increased in the fetal liver and ileum of HFD but decreased in HFSB (Fig 3a–3d). Similarly, western blotting revealed increased TNF-α expression in the fetal liver of the HFD group and decreased TNF-α expression in HFSB (Fig 3e). ZO-1, occludin and claudin-3 are essential for mucosal repair, the ZO-1, occludin or claudin-3 down-regulation may be indicate a cause of poor mucosal healing [22, 23]. The fetal ileum tight junction ZO-1 expression decreased in HFD and increased in HFSB (Fig 3f). While, there was no significant difference of occludin and claudin-3 expressions among the three groups (Fig 3f).

#### Fetal liver and ileum apoptosis

TUNEL staining, cleaved-caspase 3.

**Fig 4:**
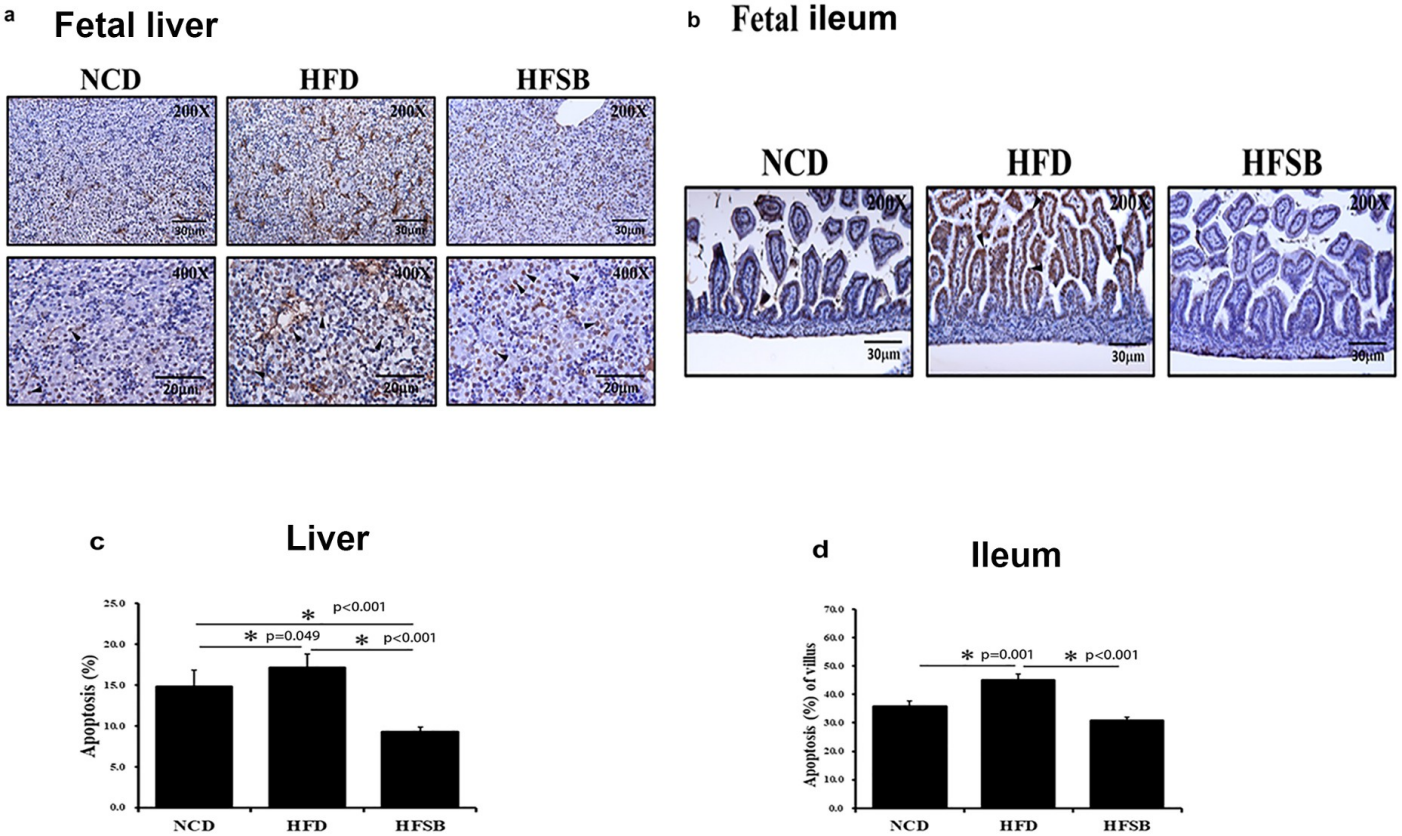
Apoptosis in the fetal liver and ileum. (a) TUNEL staining in the fetus liver and (b) fetus ileum. (c, d) Semi-quantitative analysis of fetus liver and ileum TUNEL stained positive cells. NCD: normal-chow diet, HFD: high-fat diet, HFSB: high-fat diet with butyrate supplementation during pregnancy (n = 6), * p < 0.05. Black arrowheads: positive TUNEL stained cells.

**Fig 5:**
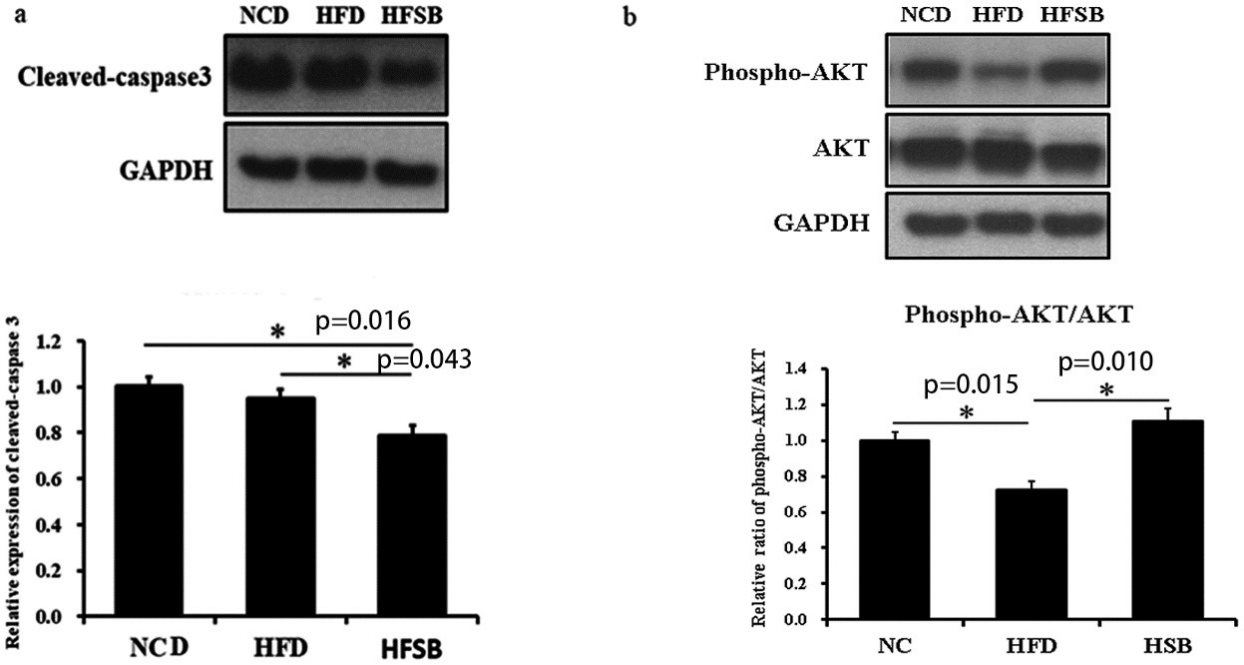
Western blotting of apoptotic protein expression in fetal liver. (a) cleaved-caspase 3, (b) phosphor-AKT/AKT. NCD: normal-chow diet, HFD: high-fat diet, HFSB: high-fat diet with butyrate supplementation during pregnancy (n = 6), * p < 0.05.

TUNEL staining indicates the activation of apoptotic pathways and was increased in the fetal liver and ileum in the HFD group but decreased in the HFSB group (Fig 4a–4d). AKT, and caspase-3 have been identified to play roles in liver cell apoptosis [24, 25]. Western blotting revealed decreased cleaved-caspase 3 expression in the fetal liver of HFSB than HFD, though it did not increase in HFD compare to NCD, the high cleaved-caspase 3 expression may be due to the anesthesia and operation process when sacrificed in NCD (Fig 5a). Phosphor-AKT was studied as an anti-apoptosis marker. The phosphor-AKT/ AKT expression decreased in HFD and increased in HFSB (Fig 5b).

#### Fetal liver oxidative stress

Lipid peroxidation and anti-oxidant enzyme.

**Fig 6:**
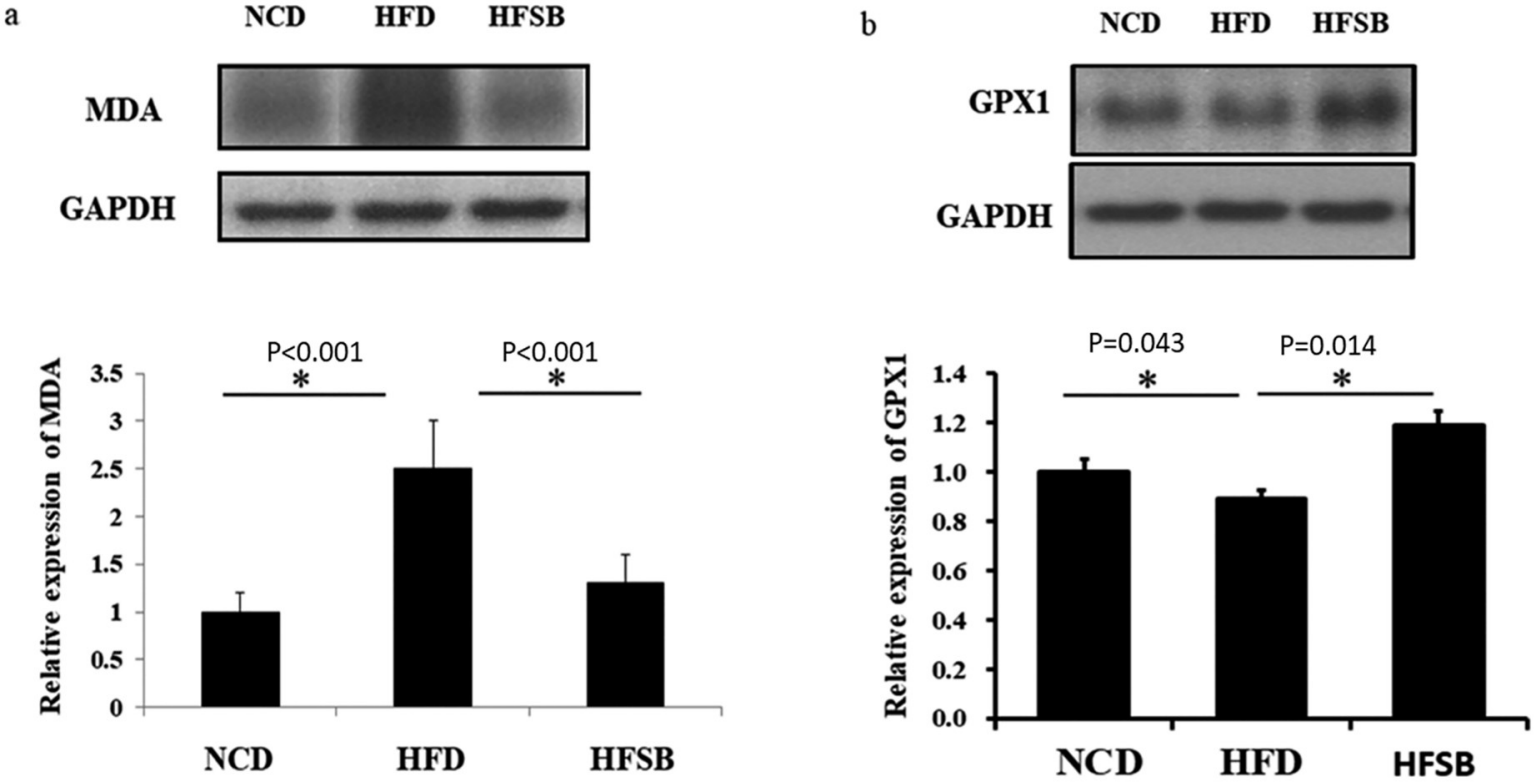
Fetal liver oxidative stress. (a) western blotting of MDA expression and (b) antioxidative stress GPX1 expression. NCD: normal-chow diet, HFD: high-fat diet, HFSB: high-fat diet with butyrate supplementation during pregnancy (n = 6), * p < 0.05.

The oxidative stress of lipid peroxidation as indicated by MDA expression was increased in the fetal liver in the HFD group but decreased in HFSB (Fig 6a). Also, antioxidant enzyme, GPX1 expression in fetal liver was decreased in HFD and increased in the HFSB group (Fig 6b).

## Discussion

Although there was no fetal liver steatosis or shortened villi in the fetal ileum after maternal HFD in this study, our results indicate that prenatal butyrate administration may alleviate HFD-induced liver steatosis and villi shortening in the ileum of pregnant rats, potentially reversing inflammation and apoptosis in fetal rat liver and ileum. The prenatal administration of butyrate reversed the HFD-induced body weight gain in pregnant rats, AST level, and fetal body weight gain. Prenatal butyrate administration also increased maternal stool butyric acid levels, increased the expression of fetal ileum tight junction proteins, and reduced the oxidative stress in the fetal liver.

It was reported that maternal HFD had offspring with higher weight gain [2, 21, 26] but no significant difference in fetal weight gain between obese mothers and non-obese mothers [3]. The fetus weight after maternal HFD and butyrate administration has not been studied. In this study, the maternal HFD increased maternal body weight and fetal body weight, which were reversed by prenatal butyrate supplementation. Raso et al. reported that the circulating levels of AST, ALT, and cholesterol increased at 6 weeks of HFD male SD rats and decreased after butyrate supplementation [12]. In this study, the AST increased in HFD pregnant mothers, but there was no change in ALT and cholesterol levels. These differences may be due to gender and pregnancy effects. Raso et al. also reported that butyrate supplementation reduced HFD male rat liver steatosis and inflammation [12]. The present study showed maternal liver lipid accumulation in prenatal HFD, which recovered in HFSB. The prenatal butyrate administration alleviated HFD-induced inflammation and apoptosis in the fetal rat liver.

Previous reports have shown that HFD led to reduced small intestinal villus length in a rodent model [27, 28]. Butyrate supplementation increased jejunal villus height and villus surface area [29]. The gut liver axis activation is changed after HFD. Similarly, the present study showed that pregnant mothers fed an HFD have shorter ileum villi, which recovered after butyrate administration. Nonetheless, no shortened villi were observed in the fetal ileum.

HFD can alter the microbiota composition [27] and prenatal HFD can also change the microbiota in the offspring’s stools [30]. HFD-driven dysbiosis has been linked to chronic low-grade intestinal inflammation and gut barrier dysfunction [28, 31]. There was lower species richness in the HFD group but this richness did not change after butyrate supplementation [31]. However, it was not possible to collect fetal stool samples for the microbiota study at this GD21 stage.

### Maternal stool SCFAs

Dysbiosis with increased intestinal permeability may induce inflammation in the liver tissue and lead to liver steatosis [32]. Microbiota-derived metabolites such as SCFAs, act as signals to the liver [32]. Although there was no difference between stool acetic acid, our study showed decreased stool butyric acid and propionic acid compared to NCD, which is compatible with dysbiosis and liver damage under HFD. It has been reported that the intake of butyrate did not change the level of SCFAs in stools [13], whereas stool butyric acid increased after butyrate supplementation compared to the HFD group in the present study.

Propionic acid is an SCFA, a common food preservative and metabolic end-product of enteric bacteria in the gut [33]. It inhibits hepatocyte oxidation and interferes with oxidative metabolism in intact hepatocytes [34], thereby affecting liver fatty degeneration [35] and promotes hepatic damage in rats [33]. Our study showed that stool propionic acid did not increase after butyrate supplementation; however, this needs to be clarified in further studies.

### Fetal liver and ileum inflammation

Many animal trials have demonstrated that maternal HFD contributes to increased fetal and offspring liver inflammation and fatty liver [36, 37]. Inflammatory cytokines may facilitate the transfer of internalized microbes from mother to offspring [26]. So, the reduced excess maternal inflammation may be a promising target for preventing adverse fetal metabolic outcomes [21]. Usually, the expression of IL-6 in hepatocytes is positively associated with the degree of inflammation [21, 38]. It was reported that HFD induced a significant increase in hepatic IL-6 mRNA, and butyrate treatment significantly prevented inflammation transcription [12]. It was also reported that up-regulation of inflammatory mediators, such as TNF-α, was found in the fatty liver [21, 39]. Butyrate supplementation suppressed several pro-inflammatory genes [15] and reduced TNF-α production [12, 13]. In our study, inflammation increased in the fetal liver as evidenced with increased IL-6, TNF-α expressions and ileum with increased IL-6 expression after maternal HFD, and the inflammation decreased with maternal butyrate supplementation. It was reported that HFD mothers developed nonalcoholic fatty liver disease in the fetal offspring [36]. A murine study showed that intestinal IL-6 expression positively correlated with intestinal permeability [40], shortened intestinal villi length after HFD with disrupted epithelium barrier function [28]. However, in this study, no liver lipid accumulation and shortened ileum villi were observed in the HFD fetus. But decreased tight junction protein expression and increased inflammation after HFD in the fetal ileum, which was reversed by butyrate supplementation. These results suggest that butyrate supplementation at this stage is more important to prevent offspring liver and ileum inflammation, as well as ileum tight junction insult.

### Fetus liver and ileum apoptosis

Xie reported an increased proportion of apoptotic cells in the intestines of HFD female mice [28] and maternal HFD increased fetal hepatic apoptosis in female Japanese macaques [41]. We found activation of apoptotic pathways in the rat fetal liver and ileum with more TUNEL staining in the HFD group which could be recovered in the HFSB group. Li reported that butyrate treatment decreased caspase-3 and caspase-9 protein levels in mammary epithelial cells [16]. Similarly, the present study showed no increase in cleaved-caspase 3 in the HFD group but it was decreased in HFSB compared to HFD. It was reported that HFD increased phosphor-AKT^S473^ in steatohepatitis [42], and Lee reported that decreased liver steatosis increased phosphor-AKT^S473^ expression [43], while Li showed that butyrate treatment increased phospho-AKT^S473^ protein levels in epithelial cells [16]. In our study, phosphor-AKT/AKT expression decreased in the fetal liver in HFD and reversed in HFSB.

### Fetal liver oxidative stress

Pregnancy with acute fatty liver is an uncommon but clinically severe hepatopathy, and oxidative stress-mediated apoptosis may be its key pathogenesis [44]. Fetal offspring from HFD mothers showed increased hepatic oxidative stress with 4-hydroxy-2-nonenal early in the third trimester [36]. Oxidative stress is the imbalance between the production of *reactive oxygen species* generated in the aerobic metabolism and their elimination by antioxidant defense enzymes [39]. The stress in the fatty liver may lead to substantial damage to cell structure include lipids and liver cirrhosis, with free radicals reacting with lipids to produce hydroperoxides, which can generate reactive intermediates, such as MDA, to cause cell death. [45–47] Fatty liver shows enhanced oxidative stress which may increase MDA with lipid peroxidation and decreased GPX [48, 49]. The supplementation of butyrate after HFD increased the antioxidants and decreased liver inflammation, as well as improving intestinal inflammation, oxidative stress, and epithelial defense barrier [12, 15]. In our study, GPX1 expression in the fetal liver decreased in HFD and increased in HFSB, with increased MDA production in the fetal liver, which recovered in HFSB. Taken together, this suggests that oxidative stress is increased in the fetal liver and antioxidative enzyme activity decreased after maternal HFD, which can be reversed with prenatal butyrate supplementation.

### What the current work add to the existing knowledge

Butyrate supplementation had a reduction of liver steatosis and inflammation in HFD fed animals [12], and had a trend to protect HFD-induced intestinal barrier impairments [31]. There were no studies investigated the effect of prenatal butyrate in the fetal liver or fetal intestine after maternal HFD. The current study revealed anti-inflammatory and anti-apoptotic effects of prenatal butyrate on the fetal liver and intestine, the decrease in ileal villous length in HFD dams was also reversed.

#### Limitations

This study has some limitations. Differences in insulin-sensitive or resistant pregnant mothers were not assessed in this research. It was difficult to collect fetal stool samples, so the status of dysbiosis could not be confirmed in the fetus. The effect of prenatal butyrate administration on offspring after delivery also requires further investigation.

In conclusion, maternal HFD can cause liver inflammation, liver steatosis, and gut microbiota dysbiosis in pregnant mothers, playing a vital role in the fetal liver via oxidative stress with inflammation. Prenatal butyrate may ameliorate the degree of inflammation, oxidative stress, and apoptosis in both the mother and fetus.

## Supporting information

S1 Raw images The original unadjusted and uncropped images for blot or gel data. (PDF)

## Abbreviations

ALT: alanine transaminase
AST: aspartate transaminase
*GPX1*: glutathione peroxidase 1
HFD: high-fat diet
HFSB: high-fat diet co-treated with butyrate during pregnancy
MDA: malondialdehyde
NCD: normal-chow diet
SCFAs: short chain fatty acids
TNF-α: tumor necrosis factor-alpha
TUNEL: TdT-mediated dUTP Biotin Nick-End Labeling
