# Supplementary material for: Butyrate ameliorates maternal high-fat diet-induced fetal liver cellular apoptosis
Source: PLoS One. 2022 Jul 6;17(7):e0270657. doi: 10.1371/journal.pone.0270657 (PMC9258878; doi:10.1371/journal.pone.0270657)

Fig.3e Manipulation of blots: cleaning up background.

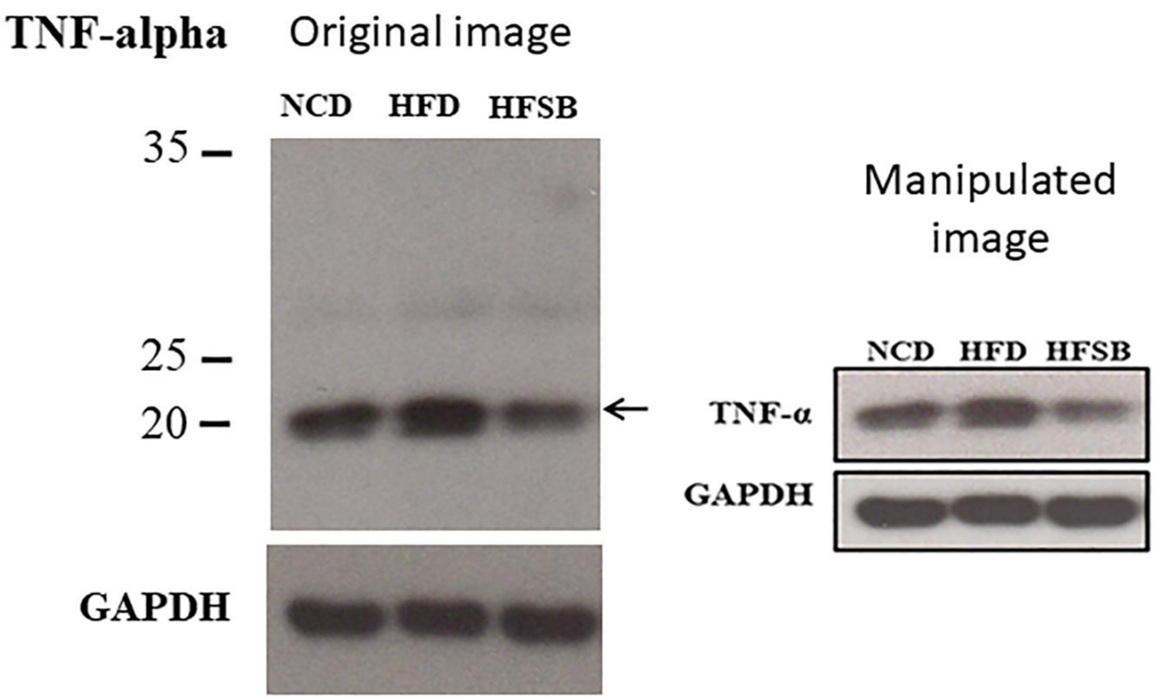

Fig. 3f Manipulation of blots: cleaning up background, brightness and contrast adjustments.

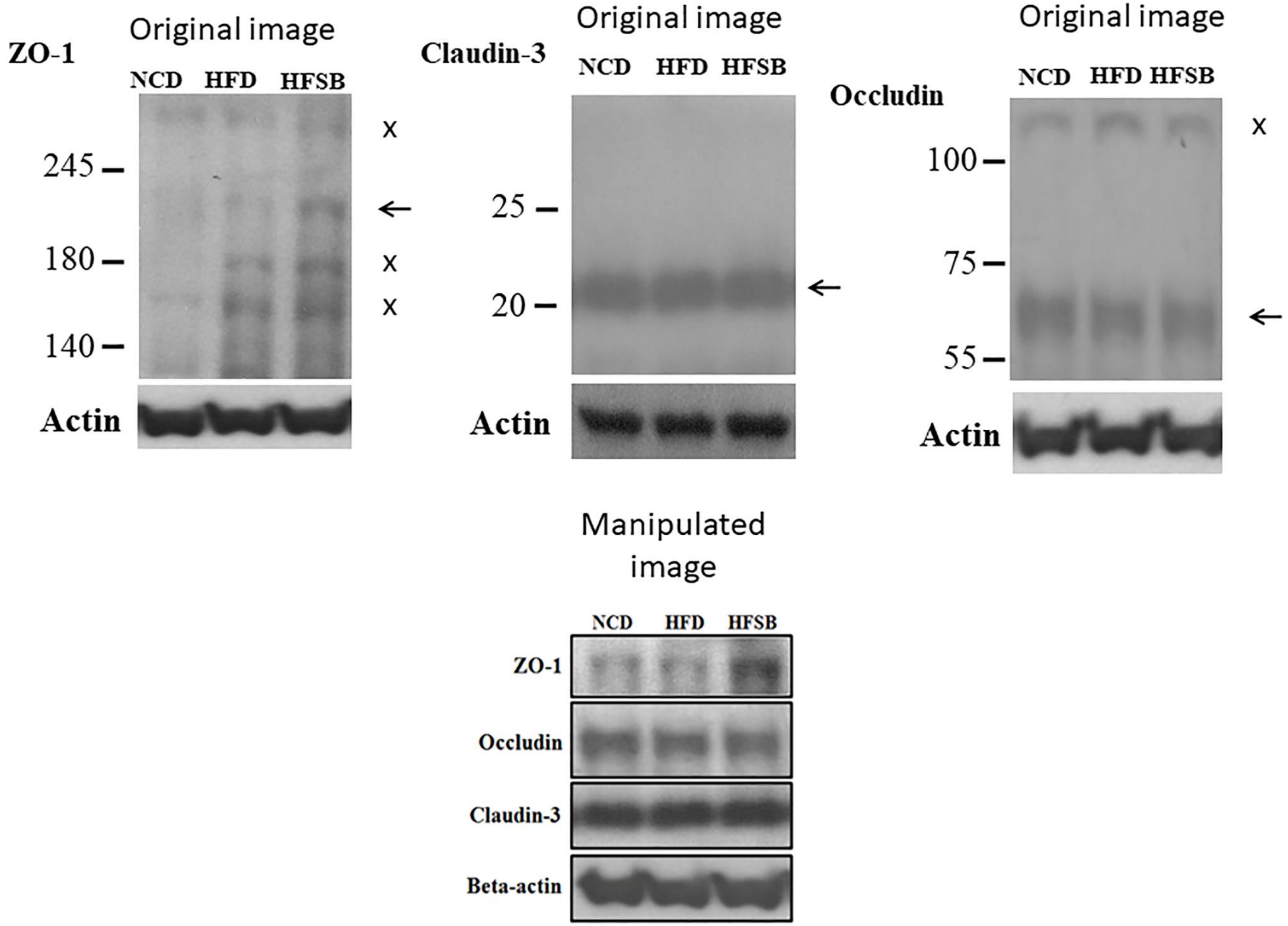

Fig. 5a Manipulation of blots: cleaning up background, brightness and contrast adjustments.

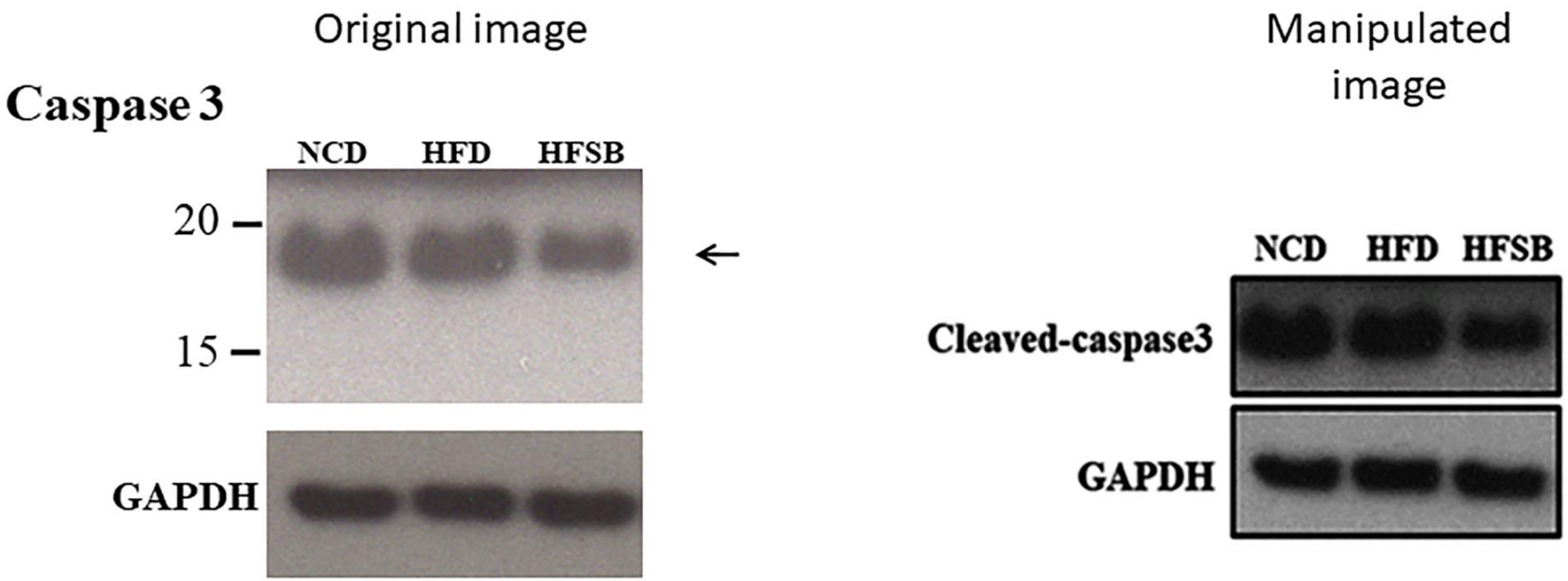

Fig. 5b Manipulation of blots: cleaning up background, brightness and contrast adjustments.

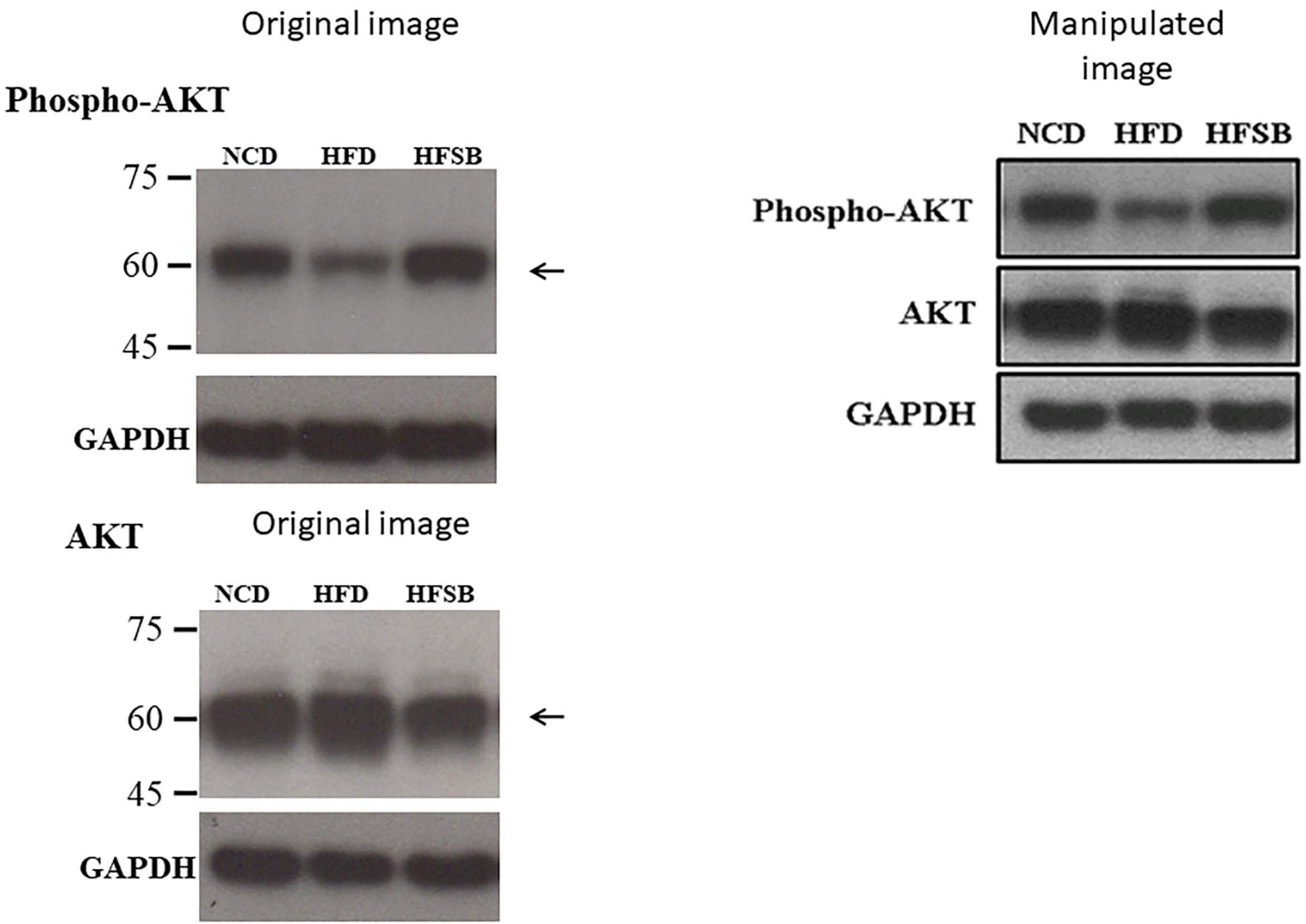

Fig. 6a Manipulation of blots: cleaning up background, brightness and contrast adjustments.

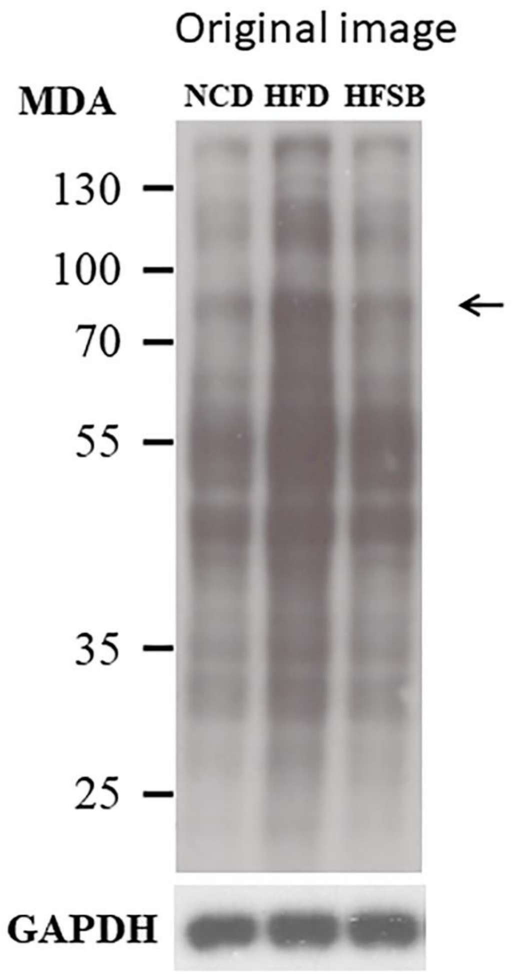

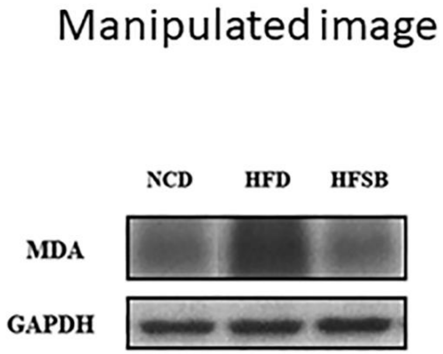

Fig. 6b Manipulation of blots: cleaning up background, brightness and contrast adjustments.

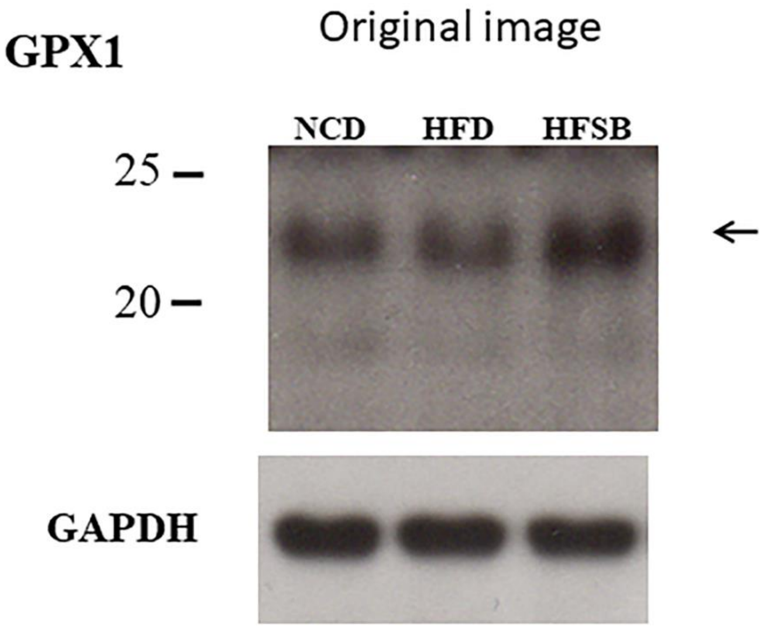

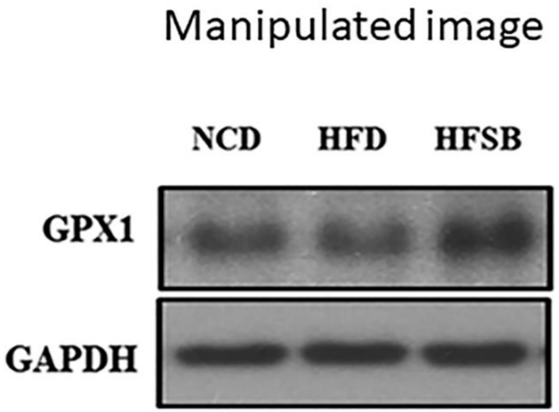

Supplement: S1 Raw images — (PDF) [file pone.0270657.s001.pdf]
